# Draft Genome Assembly of the Sheep Scab Mite, Psoroptes ovis

**DOI:** 10.1128/genomeA.00265-18

**Published:** 2018-04-19

**Authors:** Stewart T. G. Burgess, Kathryn Bartley, Edward J. Marr, Harry W. Wright, Robert J. Weaver, Jessica C. Prickett, Margaret Hughes, Sam Haldenby, Phuong Thi Le, Stephane Rombauts, Thomas Van Leeuwen, Yves Van de Peer, Alasdair J. Nisbet

**Affiliations:** a Moredun Research Institute (MRI), Edinburgh, United Kingdom; b Institute of Integrative Biology, University of Liverpool, Liverpool, United Kingdom; c Department of Plant Biotechnology and Bioinformatics, Ghent University, Ghent, Belgium; d VIB Center for Plant Systems Biology, Ghent, Belgium; e Bioinformatics Institute Ghent, Ghent University, Ghent, Belgium; f Nutriomics: Nutrition and Obesity–Systemic Approaches, Hôpital la Pitié Salpêtrière, Paris, France; g Integromics, Hôpital la Pitié Salpêtrière, Paris, France; h Department of Genetics, University of Pretoria, Pretoria, South Africa; i Department of Plants and Crops, Ghent University, Ghent, Belgium; j Fera Science Ltd., York, United Kingdom

## Abstract

Sheep scab, caused by infestation with Psoroptes ovis, is highly contagious, results in intense pruritus, and represents a major welfare and economic concern. Here, we report the first draft genome assembly and gene prediction of P. ovis based on PacBio *de novo* sequencing. The ∼63.2-Mb genome encodes 12,041 protein-coding genes.

## GENOME ANNOUNCEMENT

Sheep scab, caused by the ectoparasitic mite Psoroptes ovis, is characterized by pruritus and skin irritation and is a major welfare and economic concern for the livestock industry (1, 2). Control relies on injectable macrocyclic lactone-based endectocides and organophosphate dips, but concerns over residues, environmental contamination, and the development of resistance threaten the sustainability of this approach and have highlighted interest in developing alternative control methods (3). However, the development of novel vaccines and identification of potential chemotherapeutic targets has been hampered by the lack of transcriptomic and genomic resources for P. ovis.

Adult female P. ovis mites (∼3,200, Moredun isolate) were harvested, as previously described (4), from experimentally infested sheep. Ethical approval was obtained from the Moredun Research Institute’s local ethics committee (E03/17). Any contaminating host material was removed by extensive washing before mites were cleaned with 0.01% Triton X-100. Mites were ground under liquid nitrogen in a prechilled (−80°C) pestle and mortar, and genomic DNA (gDNA) was extracted using the SDS-proteinase K method (5). DNA integrity was assessed on an Agilent Bioanalyzer and quantified using a Qubit double-stranded DNA (dsDNA) broad-range (BR) assay kit. PacBio sequencing libraries were generated from high-molecular-weight gDNA using the PacBio SMRTbell template prep kit version 1.0 according to the manufacturer’s instructions, and sequencing was performed using 10 single-molecule real-time (SMRT) cells on the PacBio RS II platform. Genomic sequences were assembled using the Hierarchical Genome Assembly Process version 3 (HGAP3) pipeline embedded in PacBio SMRT Portal version 2.3.0.140936.p0 (6). Filtered subreads were aligned to contigs using BLASR (7), and the corrected consensus reads were generated using Quiver (6). The contaminating sequences were identified using BLASTx searches of the NCBI prokaryotic RefSeq data set, combined with tetramer frequencies and read coverage for emergent self-organizing map (ESOM) clustering, and removed from the final polished assembly, which contained 93 contigs, with an *N*_50_ value of 2,279,290 bp and an *L*_50_ value of 8 contigs (8, 9, 10). The largest scaffold was 5,538,194 bp and, as with other closely related mites, the GC content of the coding sequences was 33.6%. The total genome size was estimated to be 63.2 Mb, and 14 Gb of PacBio data provided ∼220-fold coverage.

Gene prediction was performed with EUGENE version 4.1 (11), which was pretrained on Dermatophagoides farinae (BioProject number PRJNA379991). The extrinsic data used to support the *ab initio* gene models were protein sets from Tetranychus urticae, RefSeq data (invertebrates), D. farinae predictions, and transcript data from P. ovis. The pipeline identified 12,041 predicted protein-coding genes, with an average length 1,928.21 bp, in agreement with the predicted average gene size (∼1,800 bp) for a genome of ∼63.2 Mb. The presence of core eukaryotic protein-coding genes was assessed with BUSCO version 1.22 (arthropod set) (12) with 91% of arthropod single-copy orthologs present. Predicted genes were functionally annotated by combining InterProScan (13) and reciprocal best BLAST hits, generating sensible gene function descriptions. Potential secretion signals, transmembrane helices, and other domains were predicted in PHOBIUS (14). BLAST hits against the NCBI nonredundant (nr) database (July 2016) were identified for 9,964 genes, and gene ontology (GO) assessment was performed in Blast2GO (15), resulting in the assignment of GO terms for 7,957 genes and functional annotation for 5,217 genes.

### Accession number(s).

This whole-genome shotgun project has been deposited at DDBJ/ENA/GenBank under the accession number PQWQ00000000. The version described in this paper is the first version, PQWQ01000000.
